# The role of S100A9 in the interaction between pancreatic ductal adenocarcinoma cells and stromal cells

**DOI:** 10.1007/s00262-021-03026-y

**Published:** 2021-08-10

**Authors:** Pin-Jui Kung, Ting-Yu Lai, Jerry Cao, Li-Chung Hsu, Tsai-Chen Chiang, Pu Ou-Yang, Ching-Yi Tsai, Yi-Fen Tsai, Chih-Wen Lin, Chien-Chia Chen, Meng-Kun Tsai, Yu-Wen Tien, Chih-Yuan Lee

**Affiliations:** 1 Department of Surgery, National Taiwan University Hospital and College of Medicine, National Taiwan University, No. 7, Chung-Shan South Road, Taipei, 10002 Taiwan; 2 Genome and Systems Biology Degree Program, National Taiwan University and Academia Sinica, Taipei, Taiwan; 3 Institute of Molecular Medicine, College of Medicine, National Taiwan University, No. 1 Jen-Ai Road, Sec. 1, Taipei, 10002 Taiwan; 4 Department of Surgery, Wollongong Hospital, Loftus Street, Wollongong, NSW 2500 Australia; 5 Department of Medical Research, National Taiwan University Hospital, No. 7 Chung-Shan South Road, Taipei, 10002 Taiwan; 6 Division of Gastroenterology and Hepatology, E-Da Dachang Hospital, and School of Medicine, College of Medicine, I-Shou University, No. 1, Sec. 1, Syuecheng Road, Dashu District, Kaohsiung City, 84001 Taiwan; 7 Department of Surgery, National Taiwan University Hospital, Hsin-Chu Branch, Hsin-Chu City, Taiwan

**Keywords:** S100A9, PD-L1, Pancreatic ductal adenocarcinoma, PSC

## Abstract

**Background:**

A major feature of the microenvironment in pancreatic ductal adenocarcinoma (PDAC) is the significant amount of extracellular matrix produced by pancreatic stellate cells (PSCs), which have been reported to enhance the invasiveness of pancreatic cancer cells and negatively impact the prognosis.

**Methods:**

We analyzed the data from two publicly available microarray datasets deposited in the Gene Expression Omnibus and found candidate genes that were differentially expressed in PDAC cells with metastatic potential and PDAC cells cocultured with PSCs. We studied the interaction between PDAC cells and PSCs in vitro and verified our finding with the survival data of patients with PDAC from the website of The Human Protein Atlas.

**Results:**

We found that PSCs stimulated PDAC cells to secrete S100A9, which attracted circulatory monocytes into cancer tissue and enhanced the expression of programmed death-ligand 1 (PD-L1) on macrophages. When analyzing the correlation of S100A9 and PD-L1 expression with the clinical outcomes of patients with PDAC, we ascertained that high expression of S100A9 and PD-L1 was associated with poor survival in patients with PDAC.

**Conclusions:**

PSCs stimulated PDAC cells to secrete S100A9, which acts as a chemoattractant to attract circulatory monocytes into cancer microenvironment and induces expression of PD-L1 on macrophages. High expression of S100A9 and PD-L1 was associated with worse overall survival in a cohort of patients with PDAC.

**Supplementary Information:**

The online version contains supplementary material available at 10.1007/s00262-021-03026-y.

## Introduction

Pancreatic cancer is one of the most lethal cancers worldwide. Approximately 80% of pancreatic cancers originate from exocrine cells, and the majority of pancreatic cancers are pancreatic ductal adenocarcinomas (PDACs). A major focus in the field of cancer research has been the cancer cells themselves; however, cancers do not simply consist of malignant cells alone. One of the features of PDAC is the presence of an extensive desmoplastic reaction consisting of pancreatic stellate cells (PSCs), immune cells, fibroblasts, endothelial cells and extracellular matrix. Studying the nature of the tumor microenvironment may be as important for future cancer therapies as studying the cancer cell itself. Different types of stromal cells in the tumor microenvironment can be recruited by cancer cells to support their growth and facilitate metastasis [1]. Cancer cells do not act in isolation, and their growth is determined not only by themselves but also by their interactions with the microenvironment[2]. As malignancy progresses, the surrounding microenvironment continues to interact with cancer cells and delivers signals that promote growth, foster chemoresistance and ultimately facilitate distant metastasis[3, 4]. Macrophages, inflammatory cells and other stromal cells can also be attracted and activated by signals generated in the cancer microenvironment.

A major feature of the microenvironment in PDAC is the significant amount of extracellular matrix produced by stromal cells [5, 6]. PSCs are likely to produce the extracellular matrix and interstitial connective tissue components, including collagen type I and fibronectin[7]. Several studies have reported that PSCs enhance the invasiveness and proliferation of pancreatic cancer cells and negatively impact prognosis [8, 9]. Therefore, we tried to study the interaction between PDAC cells and PSCs. There are publicly available microarray data deposited in the Gene Expression Omnibus (GEO) database at http://www.ncbi.nlm.nih.gov/geo/, and we analyzed the dataset GSE36775, in which the gene expression of pancreatic adenocarcinoma cells cocultured with or without PSCs was studied. We also analyzed the GSE9350 dataset, in which the gene expression of pancreatic cancer cell lines with metastatic and growth potential was explored. After combining the results of the analyses of the two datasets (GSE36775 and GSE9350), we found that the gene expression of S100 calcium binding protein A9 (S100A9) was increased in both datasets.

S100A9, a member of the S100 family of calcium binding proteins, has been detected in infiltrating macrophages in rheumatoid arthritis and other inflammatory conditions [10, 11]. S100A9 can be found in cells of myeloid origin, such as monocytes, neutrophils and dendritic cells [12]. After secretion, S100A9 acts as a chemoattractant to recruit inflammatory cells into the surrounding microenvironment [13–15]. Secreted S100A9 protein has also been reported to play a role in the establishment of a favorable environment for cancer growth [16, 17]. It was indicated that S100A9 protein secreted by myeloid cells within primary cancers and metastatic sites promotes the accumulation of more myeloid cells, which plays an important role in modulating tumor progression and the formation of premetastatic niches at metastatic sites [17–20]. Tumor-associated macrophages interact with cancer cells and contribute to cancer aggressiveness as an integral part of the tumorigenic pathway[21]. However, the expression of S100A8 and S100A9 in macrophages is lost rapidly after their differentiation from monocytes [16, 22].

Immune surveillance is the first-line defense to identify and eliminate cancer cells, which have developed numerous strategies to evade immune surveillance mechanisms and continue neoplastic progression. Immune checkpoints include several costimulatory and inhibitory signals of immune cells for the prevention of autoimmunity. However, these machineries can be hijacked by cancer cells to evade immune surveillance [23, 24]. Programmed death-ligand 1 (PD-L1) overexpression has been reported in infiltrating immune cells in PDAC, and increased PD-L1 expression is associated with poor survival [25].

In this study, we found that PSCs stimulate pancreatic cancer cells to secrete S100A9, which initiates the attraction of circulatory monocytes into cancer tissue. When analyzing the correlation of S100A9 and PD-L1 expression with clinical outcomes of patients with PDAC, we ascertained that high expression of S100A9 and PD-L1 was associated with worse overall survival than low expression of these proteins in a cohort of patients with PDAC.

## Materials and methods

### Microarray data analysis

The microarray datasets used in this study, GSE36775 and GSE9350, were downloaded from Gene Expression Omnibus (GEO), which can be accessed at http://www.ncbi.nlm.nih.gov/geo/. We analyzed the dataset GSE36775, which contained gene expression data of pancreatic adenocarcinoma cells cocultured with or without PSCs. We also analyzed the dataset GSE9350, which contained gene expression data of pancreatic cancer cell lines with high metastatic and growth potential under normoxic growth conditions. We analyzed these datasets with the web-based tool GEO2R at https://www.ncbi.nlm.nih.gov/geo/geo2r/ to obtain adjusted p-values for multiple comparisons with the Benjamini & Hochberg method and log2-transformed fold change values. The microarray datasets were deposited by the original investigators, and individual quality control measures were reported in the original references [9, 26].

### Patients

In this study, we retrospectively collected specimens for immunofluorescence staining from 16 patients with PDAC varying from stage IIa to stage IV who received operative intervention at National Taiwan University Hospital between 2001 and 2008. The study was approved by the institutional review boards (201705130RINC).

### Cell culture

Human PSCs were purchased from ScienCell Research Laboratories, Inc. (Carlsbad, CA). The other cell lines were obtained from the Bioresource Collection and Research Center (Hsin-chu, Taiwan). The human pancreatic cancer cell lines AsPC-1, HPAF-II and PANC-1 were cultivated in RPMI 1640 medium (Thermo Fisher), MEM (Thermo Fisher) and DMEM (Thermo Fisher) supplemented with 10% fetal bovine serum (FBS) (Thermo Fisher). PSCs were cultured in SteCM medium (ScienCell™, CA, USA) containing 2% FBS, essential and nonessential amino acids, vitamins, organic and inorganic compounds, hormones, growth factors and trace minerals. The human monocyte and lymphocyte-like cell lines THP-1 and U937 (CRL-1593.2) were maintained in RPMI 1640 medium with 10% FBS. All cell lines were incubated at 37 °C with 5% CO_2_. Pancreatic cancer cells (5 × 10^5^) were seeded in 6-well plates and cocultured with or without 5 × 10^5^ PSCs on 0.4 μm PET hanging inserts (Merck). All cell lines were tested for mycoplasma contamination by the MycoAlert™ PLUS Mycoplasma Detection Kit (LT07-701, Lonza).

### Plasmids and transfection

We seeded 1 × 10^6^ HEK293 cells in a 6-well plate one day before transfection. On the next day, we transfected plasmids into HEK293 cells with Lipofectamine™ 3000 Reagent (Thermo Fisher) according to the manufacturer's protocol and collected the cell pellets for further analysis 72 h after transfection.

### *shRNA*-mediated gene silencing

Short hairpin RNA (*shRNA*) constructs encoding shRNAs against human S100A9 in pLKO-puro vectors were obtained from the National RNAi Core Facility, Academia Sinica, Taiwan. The clone identity numbers were TRCN0000415789 for *S100A9* shRNA#1 (*shS100A9#1*), TRCN0000425882 for *shS100A9#2*, TRCN0000419023 for *shS100A9#3*, TRCN0000433650 for *shS100A9#4* and TRCN0000072243 for *shControl.* We transfected shRNAs into cells with Lipofectamine™ 3000 Reagent (Thermo Fisher) according to the manufacturer's protocol.

### Conditioned media preparation

Pancreatic cancer cells were counted and seeded in 6-well plates with RPMI medium. After 24 h, cells were washed twice with phosphate-buffered saline (PBS) and incubated in RPMI medium for an additional 24 h at 37 °C. Pancreatic cancer cells (5 × 10^5^) were seeded in 6-well plates and co-cultured with or without 5 × 10^5^ PSCs on 0.4 μm PET Millicell® hanging inserts (Merck) for 24–72 h. The conditioned medium was generated by centrifugation at 1000 g for 5 min to separate cell debris.

### Transwell migration assays

THP-1 (2 × 10^5^) or U937 (2 × 10^5^) cells were seeded inside hanging inserts (Millicell® hanging inserts, 8.0 μm, Merck) suspended over wells with conditioned media in a 12-well plate. The conditioned media were obtained from 5 × 10^5^ pancreatic cancer cells (AsPC-1, HPAF-II or PANC-1 cells) cocultured with 5 × 10^5^ PSCs in a Millicell® hanging insert (0.4 μm polyethylene terephthalate membrane, Merck) in 6-well plates for 24 h. The conditioned media were centrifuged at 1000 g for 5 min before being employed in further experiments. The migrated cells were counted in four different fields, and the experiments were repeated in triplicate. Primary human monocytes (2.5 × 10^5^) were seeded inside hanging inserts (8.0 μm, Merck) suspended in the wells with or without S100A9 stimulation in a 24-well plate.

### Precipitation of proteins in the culture medium

The culture medium was precipitated by adding 20% trichloroacetic acid (TCA), mixed well and stored in a refrigerator at −20 °C overnight, and then centrifuged at 12,000 g for 30 min. After centrifugation, the pellet was washed twice with acetone, and lysis buffer was added to the dry pellet for further analysis.

### Antibodies and recombinant proteins

Antibodies against S100A9 (PA5-19,075, 1:1000) and Flag (PA1-984B, 1:1000) were obtained from Thermo Fisher, and anti-GAPDH antibodies (#5174, 1:1000) were obtained from Cell Signaling Technology (Danvers, MA). The anti-PD-L1 monoclonal antibody (29E.2A3) was obtained from BioLegend (San Diego, CA). Allophycocyanin (APC)-conjugated anti-mouse IgG antibodies (115–605-166) were obtained from Jackson ImmunoResearch Inc. (West Grove, PA). Human S100A9 recombinant proteins were obtained from R&D (9254-S9-050, R&D systems, Minneapolis, MN), and human S100A8/S100A9 heterodimer proteins were from biolegend (753,406, BioLegend, San Diego, CA).

### Immunoblotting analysis

We collected and washed cells twice with PBS, added RIPA buffer containing protease inhibitor into each tube and centrifuged the samples at 12,000 g for 30 min. The protein concentration was measured using a Pierce BCA protein assay kit (Thermo) according to the manufacturer's protocol. Protein extracts (20–40 μg) and the concentrated product of each supernatant (10 µl) were run on 12% SDS polyacrylamide gels, and separated proteins were transferred to a PVDF membrane for further detection.

### mRNA purification and quantitative RT-PCR

We extracted mRNA with a NucleoSpin RNA Kit (Macherey–Nagel, GmbH & Co., KG, **Düren**) and synthesized cDNA with a PrimeScript™ RT Reagent Kit (Takara, Japan) according to the manufacturers’ instructions. Real-time quantitative PCR (qPCR) was performed with Maxima SYBR Green/ROX qPCR Master Mix (Thermo Scientific, Rockford, IL) on a QuantStudio 3 instrument (Thermo Scientific) according to the manufacturer’s recommendations. All qPCR values were normalized to cyclophilin mRNA levels as an internal control to obtain the relative values. All experiments were performed in triplicate. The primer sequences are shown in Supplementary Table 3.

### Differentiation of U937 monocytes into macrophages

The human monocytic cell line U937 was maintained in RPMI-1640 medium with 10% FBS (Gibco®) and penicillin/streptomycin (Gibco®). Cells were maintained at 37 °C in a humidified 5% CO_2_ atmosphere. For differentiation, U937 cells were grown overnight on a 12-well plate at a density of 2 × 10^5^ cells per well. Cells were then incubated with 25 ng/ml phorbol-12-myristate-13-acetate (PMA) (Sigma-Aldrich, St. Louis, MO) for 48 h and washed with Dulbecco's phosphate-buffered saline (DPBS) to remove nonadherent cells. Cells were incubated in RPMI-1640 medium for 24 h before they were ready for further assays.

### Human monocyte differentiated macrophages

Human peripheral blood monocytes were purchased from STEMCELL Technologies Inc. (catalog no. 70034, Vancouver, Canada). Monocytes were counted and then seeded (1 × 10^6^ /ml) in 24 well non-treated culture plates (catalog no. 1820-024, Iwaki, Asahi Glass Co.). After 50 min, non‐adherent cells were removed, and those adherents were cultured in RPMI-1640 medium (Invitrogene) supplemented with 2 mM L‐glutamine, 100 U/ml penicillin, 100 µg/ml streptomycin and 10% Human AB type serum (H4522, Sigma-Aldrich) at 37°C (5% CO_2_) for 7 days. Culture media was changed on day 3 and day 6.

### Immunofluorescence

Wax-embedded human pancreas blocks were cut into 5-μm-thick sections and dewaxed, and antigens were retrieved by Trilogy™ (Cell Marque) treatment at 121 °C for 3 min. Sections were blocked with 3% BSA for 30 min and then incubated overnight with appropriate primary antibodies (anti-S100A9, 1:200, 26,992–1-AP, Proteintech; anti-α-SMA, 1:200, MMS-466S, BioLegend). After washing in DPBS, the pancreas sections were further incubated with fluorophore-conjugated secondary antibodies (Alexa Fluor 488, 1:300, Jackson ImmunoResearch. Alexa Fluor 594, 1:300, A-21207, Thermo Fisher Scientific) for 1 h. Hoechst 33,342 Solution (1:10,000, Thermo Fisher Scientific) was used for staining nuclei. Images were captured by TissueFAXS PLUS (TissueGnostics, Vienna, Austria) and quantified by the StrataQuest Analysis System (TissueGnostics).

### Flow cytometry

PD-L1 expression levels on differentiated U937 macrophages and human monocyte-derived macrophages were measured by flow cytometry. Briefly, cells were incubated with anti-human PD-L1 monoclonal antibodies (29E.2A3, BioLegend, San Diego, CA), followed by staining with allophycocyanin (APC)-conjugated anti-mouse IgG antibodies (115–605-166, Jackson ImmunoResearch Inc. West Grove, PA). Stained cells were processed on a BD *FACSVerse* cell analyzer, and flow cytometry data were analyzed by FlowJo™ Software for Windows Version 10 (Becton, Dickinson and Company, Ashland).

### Statistical analysis

We performed statistical analysis with STATA 14 for Windows (StataCorp, College Station, TX). A p-value less than 0.05 was considered statistically significant.

## Results

### Microarray data analysis

The publicly available microarray datasets GSE36775 and GSE9350 were downloaded from Gene Expression Omnibus (GEO), which can be accessed at https://www.ncbi.nlm.nih.gov/geo/. We retrieved the microarray dataset GSE36775, which included gene expression data of pancreatic adenocarcinoma cells exposed to stromal PSCs in a 3D gel matrix, and analyzed the dataset with GEO2R at https://www.ncbi.nlm.nih.gov/geo/geo2r/ (Supplementary Table 1). We also analyzed the dataset GSE9350, which included gene expression data of pancreatic cancer cell lines with high metastatic and growth potential under normoxic conditions (Supplementary Table 2). After combining the results of the analyses of the two datasets (GSE36775 and GSE9350), we found that the gene expression of S100A9 was increased in both datasets. According to the analyses of the two microarray datasets, we hypothesized that PSCs might stimulate pancreatic cancer cells to secrete S100A9 and influence the progression of pancreatic cancer.

### The expression of S100A9 in pancreatic cancer cells is induced by PSCs

**Fig. 1:**
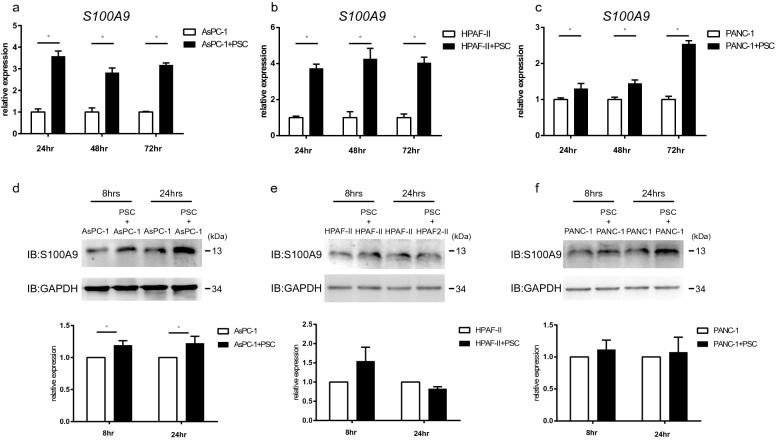
Expression of S100A9 in PDAC cells was enhanced when they are cocultured with PSCs. The protein and gene expression of S100A9 was upregulated when PSCs were cocultured with PDAC cell lines, including AsPC-1 cells (**a** and **d**), HPAF-II cells (**b** and **e**) and PANC-1 cells (**c** and **f**). In the coculture system, PDAC cells were cultured in a 6-well plate with or without PSCs in Millicell® 0.4 μm-pore hanging inserts (Merck) for the indicated time points. All experiments were performed in triplicate

To validate the results of the microarray analysis, we used a coculture system with Millicell® hanging cell culture inserts (polyethylene terephthalate insert with a pore size of 0.4 μm, Merck) to examine the interaction between pancreatic adenocarcinoma cell lines and PSCs. For coculture with hanging inserts, we cultured pancreatic cancer cell lines, including AsPC-1, HPAF-II and PANC-1 cells, in 6-well plates and seeded PSCs on the hanging inserts. The gene expression of *S100A9* in AsPC-1, HPAF-II and PANC-1 cells cocultured with PSCs in the coculture system was increased compared to that in cells cultured alone (Fig. 1). The protein level of S100A9 in pancreatic cancer cell lines was also increased when the cells were cocultured with PSCs in the coculture system (Fig. 1d–f).

### PDAC cells cocultured with PSCs enhance the migration of monocytes

**Fig. 2:**
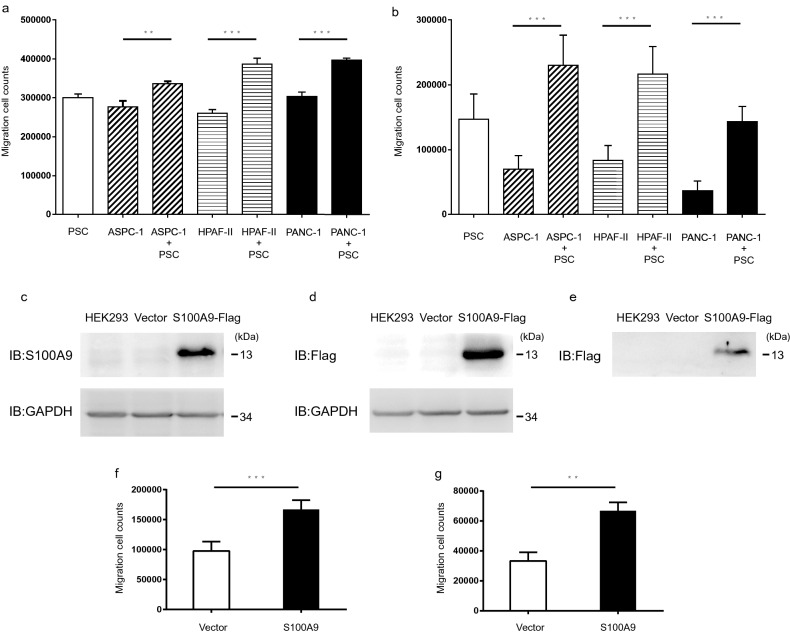
S100A9 in conditioned media from PDAC cells cocultured with PSCs enhanced the migration of monocytes. In transwell migration assays, THP-1 (**a**) or U937 (**b**) cells were cultured inside hanging inserts (8.0 μm, Merck) for 24 h in conditioned media from AsPC-1, HPAF-II and PANC-1 cells cocultured with or without PSCs. The migrated cells were counted in four fields for each sample, and the experiments were performed in triplicate. The migration of THP-1 (**a**) or U937 (**b**) cells was enhanced in the conditioned media from pancreatic cancer cell lines cocultured with PSCs for 24 h. We constructed a S100A9 plasmid with a Flag tag at the C terminus in the pcDNA3.0 vector and transfected the S100A9-Flag vector into HEK293 cells. The expression of S100A9 in HEK293 cells was verified by immunoblot with an anti-S100A9 antibody (**c**) and an anti-Flag antibody (**d**). The empty vector served as a control. Flag-tagged S100A9 protein was detected in the culture medium by immunoblot after precipitation with trichloroacetic acid (**e**). In the transwell migration assays, we placed conditioned media from HEK293 cells transduced with the S100A9 expression vector or control in the culture plates and seeded monocyte cell lines, including THP-1 or U937 (2 × 10^5^) cells, in the hanging inserts, followed by incubation for 24 h. We calculated the numbers of migrated THP-1 (**f**) and U937 (**g**) cells. We found that conditioned media with S100A9 enhanced the migration of THP-1 and U937 cells. The migrated cells were counted in four different fields, and the experiments were repeated in triplicate. *P*-values were obtained by t-test: **p < 0.005, ***p < 0.001

According to published reports, S100A9 can induce chemotaxis of immune cells [27, 28]. Therefore, we aimed to determine whether the secretion of S100A9 from pancreatic cancer cells induced by PSCs could induce the migration of monocytes. In a transwell cell migration assay (Millicell® hanging inserts, 8.0 μm, Merck), we placed THP-1 (2 × 10^5^) or U937 (2 × 10^5^) cells inside the hanging inserts (8.0 μm, Merck) and suspended the hanging inserts in the wells of culture plates containing conditioned media from pancreatic cancer cell lines cocultured with or without PSCs. We found that the conditioned media from pancreatic cancer cell lines cocultured with PSCs enhanced the migration of monocytes (Fig. 2a and b) in the transwell migration assay.

### S100A9-enriched conditioned medium induces the migration of monocytes

To examine whether S100A9 was the factor responsible for the chemotaxis of monocytes, we constructed a S100A9 vector with a Flag tag at the C terminus in a pcDNA3.0 vector and transduced the S100A9-Flag vector into HEK293 cells. We verified the expression of S100A9 in HEK293 cells by Western blot with an anti-S100A9 antibody (Fig. 2c) and an anti-Flag antibody (Fig. 2d). In the culture medium from HEK293 cells transduced with the S100A9-Flag vector, we also detected the secretion of S100A9-Flag by Western blot (Fig. 2e). In transwell migration assays, we placed conditioned media from HEK293 cells transduced with S100A9-Flag vector or control vector (empty vector only) in the culture plates and seeded monocyte cell lines, including THP-1 or U937 cells, in the hanging inserts (Millicell® hanging inserts, 8.0 μm, Merck). After 24 h, we calculated the number of migrated THP-1 (Fig. 2f) and U937 (Fig. 2g) cells. We found that conditioned media with S100A9 enhanced the migration of THP-1 and U937 cells.

**Fig. 3:**
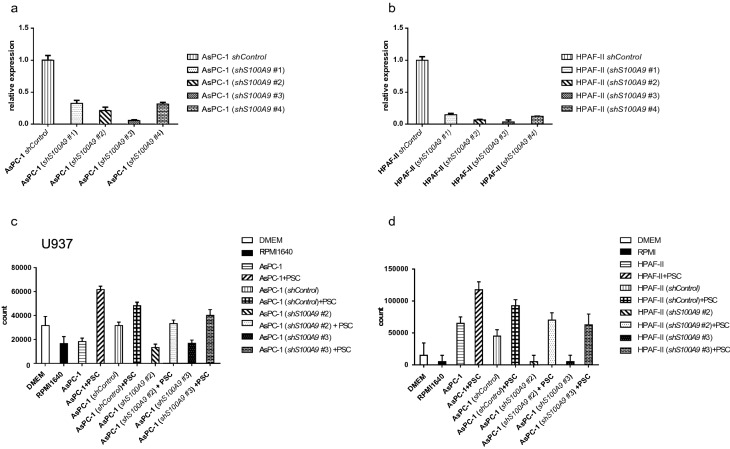
Knockdown of *S100A9* in PDAC cells affected the migration of monocytes in transwell migration assays. The *S100A9* mRNA levels in AsPC-1 (**a**) and HPAF-II (**b**) cells after knockdown of *S100A9* with shRNA are shown. The mRNA level was determined by quantitative real-time PCR. We examined transwell migration of U-937 cells (1 × 10^5^) cultured in conditioned medium from AsPC-1 (**c**) or HPAF-II (**d**) cells with either knocked down or normal *S100A9* expression cocultured with or without PSCs. We found that conditioned medium from PDAC cells cocultured with PSCs enhanced the migration of U-937 cells. The conditioned medium from PSCs cocultured with *S100A9*-knockdown AsPC-1 (**c**) and HPAF-II (**d**) cells induced less attraction of U-937 cells than the conditioned medium from PSCs cocultured with control cells. DMEM: Dulbecco's modified Eagle’s medium, RPMI 1640: Roswell Park Memorial Institute

To further verify the function of S100A9 in monocyte chemotaxis, we knocked down *S100A9* expression with shRNA in PDAC cell lines, including AsPC-1 (Fig. 3a) and HPAF-II cells (Fig. 3b). The *shluciferase* construct was used as a knockdown control in this assay.

We found that conditioned medium from PDAC cells cocultured with PSCs enhanced the migration of U-937 cells. The conditioned medium from PSCs cocultured with *S100A9*-knockdown AsPC-1 (Fig. 3c) and HPAF-II (Fig. 3d) cells induced less attraction of U-937 cells than the conditioned medium from PSCs cocultured with knockdown-*luciferase* control cells. In summary, PDAC cells cocultured with PSCs enhanced the expression of S100A9, which modulated the migration of monocytes. In contrast, S100A9-knockdown PDAC cells cocultured with PSCs attenuated monocyte chemotaxis.

### S100A9 induces surface expression of PD-L1 in monocytes

**Fig. 4:**
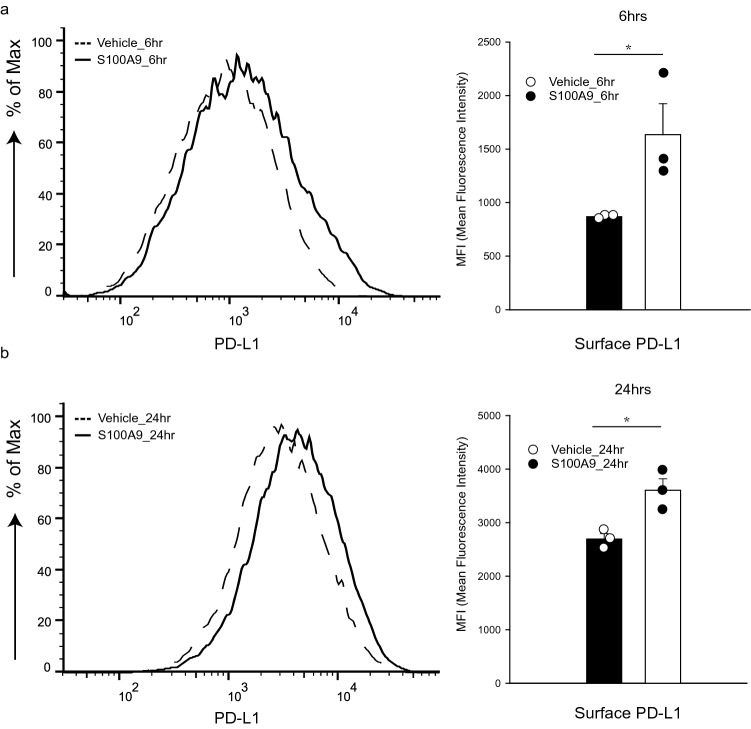
The surface expression of PD-L1 in monocyte-derived macrophages was increased after stimulation with S100A9. We treated PMA differentiated U937 cells with IFN-γ along with recombinant S100A9 and detected the surface expression of PD-L1 with flow cytometry. We found that treatment of IFN-γ-primed U937 cells with S100A9 protein for 6 (**a**) and 24 (**b**) hours enhanced the surface expression of PD-L1. We performed the experiments in triplicate. *P*-values were determined by *t*-test: *p < 0.005

In addition to its chemotactic effect, S100A9 has been reported to be the endogenous ligand of Toll-like receptor 4 (TLR4) and to induce surface expression of PD-L1 in primary hematopoietic cells [29, 30]. High expression of PD-L1 in PDAC was associated with poor prognosis [31]; however, the mechanism that regulates the expression of PD-L1 in PDAC is not clear. To verify the effect of S100A9 on the expression of PD-L1 on monocytes, we treated U937 cells with interferon gamma (IFN-γ) along with recombinant S100A9 homodimers and detected the surface expression of PD-L1 with flow cytometry. We found that treatment with recombinant S100A9 in IFN-γ-primed U937 cells for 6 (Fig. 4a) and 24 h (Fig. 4b) enhanced the surface expression of PD-L1. It was reported that S100A9 may also form heterodimers with S100A8 [32]. We treated IFN-γ-primed U937 cells with recombinant S100A8/S100A9 heterodimers, and detected the surface expression of PD-L1 with flow cytometry. We found that treatment of IFN-γ-primed U937 cells with S100A8/S100A9 heterodimers did not induce significant changes in the surface expression of PD-L1 in comparison with vehicle control (Supplementary Fig. 2).

### S100A9 induces migration of primary human monocytes and enhances surface PD-L1 expression in primary human monocyte-derived macrophages

**Fig. 5:**
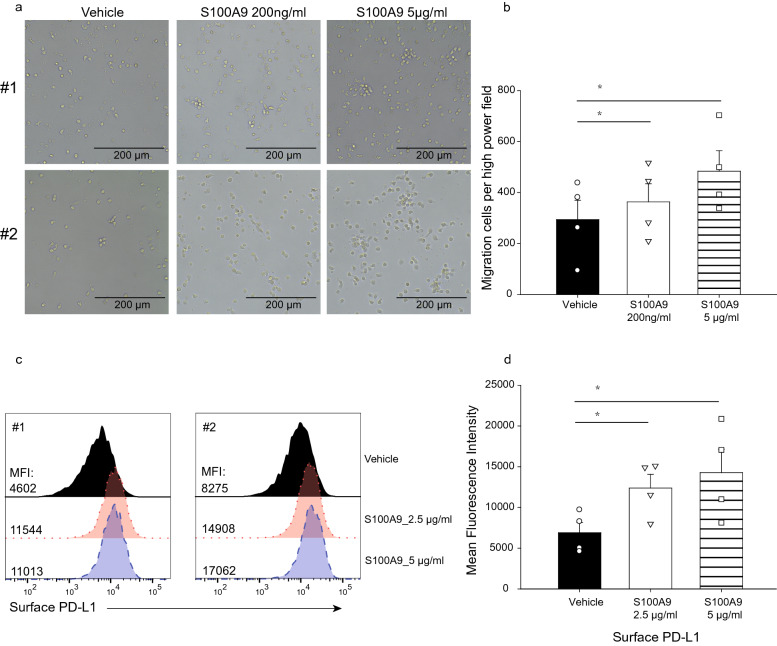
S100A9 induces migration of primary human monocytes and enhances surface PD-L1 expression in primary human monocyte-derived macrophages. (**a**) We used transwell migration assay to examine the influence of S100A9 on the migration of primary human monocytes and showed representative figures from 2 different batches (#1 and #2). Human monocytes were cultured inside hanging inserts (8.0 μm, Merck) suspended in the wells with or without S100A9 stimulation in 24-well plates for 36 h. (**b**) Column diagram indicated the mean migrated cell counts of 4 random high power microscope fields and the results from 4 independent transwell migration experiments. (*p < 0.05, paired t-test) (**c**) We examined surface PD-L1 expression in primary human monocyte-derived macrophages from different batches (#1 and #2) after treatment with S100A9. We treated human monocyte-derived macrophages with recombinant S100A9 for 24 h and detected surface expression of PD-L1 with flow cytometry. (**d**) Column diagram indicated the mean fluorescence intensity (MFI) of surface PD-L1 and the results of statistical analysis. Quadruplicate experiments were performed independently. (*p < 0.05, paired t-test)

In order to further validate the role of S100A9 in the migration of monocytes, we used primary human monocytes isolated from peripheral blood (# 70,034, STEMCELL Technologies Inc. Vancouver) to examine the influence of S100A9 on the migration of human monocytes. Consistent with the results using monocyte cell lines, we found that S100A9 would induce migration of primary human monocytes in transwell migration assay (Fig. 5a and b). We also validated that surface expression of PD-L1 in primary human monocyte-derived macrophages would be enhanced after treatment with S100A9 (Fig. 5c and d).

### S100A9-expressing cells are located near PSCs in pancreatic cancer samples

**Fig. 6:**
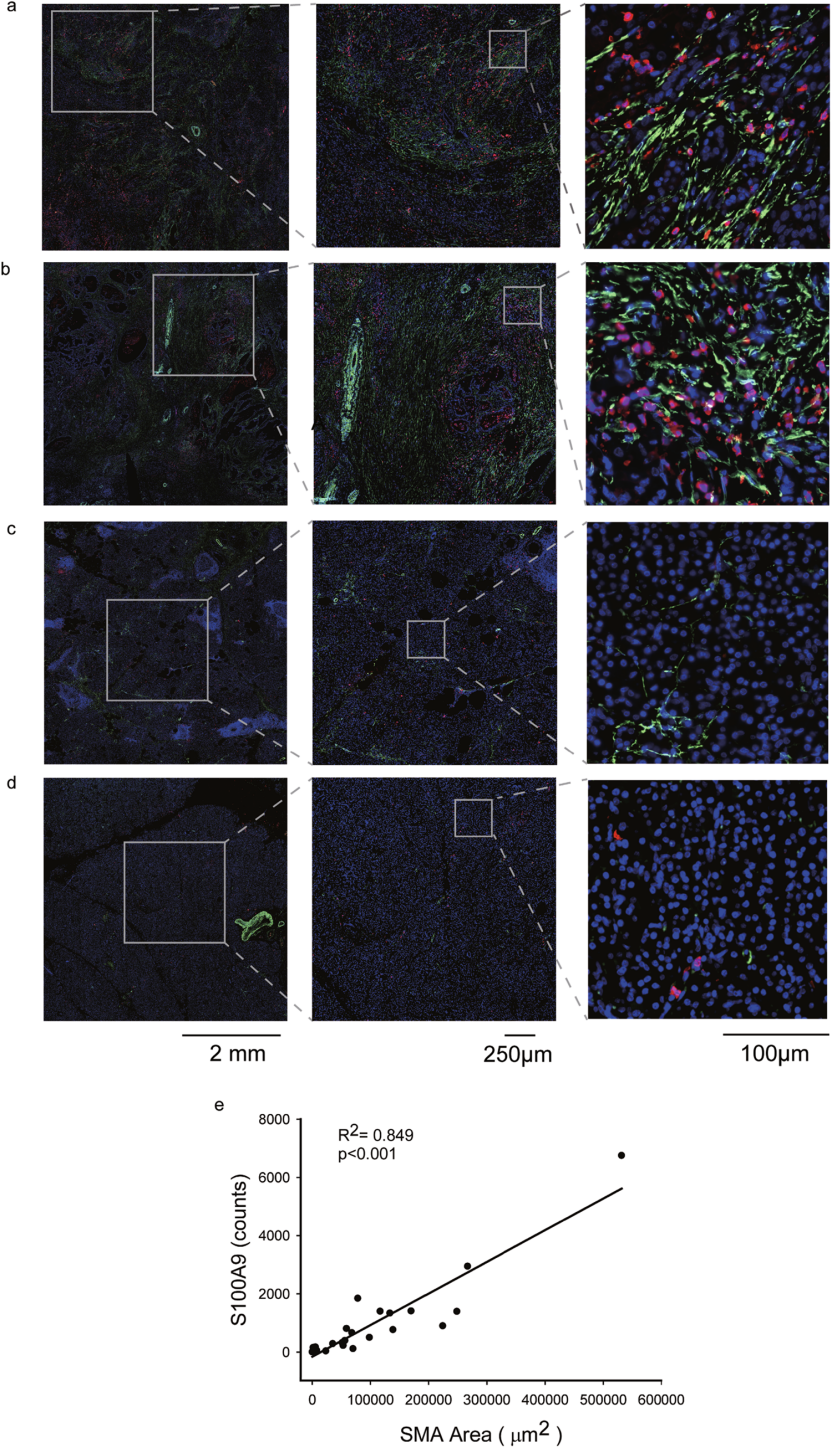
Immunofluorescence analysis of S100A9 and stellate cells in human PDAC tissue. Using immunofluorescence, S100A9-expressing cells (red) were found to be located near cells expressing α-SMA (green), a marker for activated PSCs, in specimens from PDAC patients with metastasis (**a** and **b**). Less PSC infiltration correlated with fewer S100A9-expressing cells in the specimens from patients with PDAC without metastasis (**c** and **d**). We used Hoechst 33,342 for nuclear counterstaining (blue). (**e**) The correlation between the number of cells expressing of S100A9 and the number of cells expressing α-SMA in patients with PDAC was significant in the regression model (*R*^2^ = 0.849, *p* < 0.001). Each spot represents staining of a specimen from one patient (*N* = 22)

Specimens from 22 patients with PDAC were obtained and stained for S100A9 and α-smooth muscle actin (αSMA), a differentiation marker for activated PSCs, with double immunofluorescence staining. As can be seen in the representative figures, we found that S100A9-expressing cells were located near PSCs in the specimens from patients with PDAC with metastasis (Fig. 6a and b). High levels of PSC infiltration were accompanied by increased numbers of nearby S100A9-expressing cells (Fig. 6a and b), whereas less PSC infiltration correlated with fewer S100A9-expressing cells in specimens from patients with PDAC without metastasis (Fig. 6c and d). The results suggest that PSCs can induce surrounding cells to produce S100A9 or attract S100A9-expressing myeloid cells. To study the correlation between the expression of S100A9 and α-SMA (as determined by the immunofluorescence staining) in the specimens from the 16 patients with PDAC, we scanned the whole area of slides subjected to double immunofluorescence staining (Fig. 6a–d) with the TissueFAXS system (TissueGnostics, Vienna, Austria) to avoid sampling error on the slides. Then, we quantified the scanning results of the slides with the StrataQuest Analysis System (TissueGnostics). The correlation between the numbers of cells expressing S100A9 and the numbers of cells expressing α-SMA in samples from patients with PDAC was significant in regression models (*R*^**2**^ = 0.849, *p* < 0.001). Each spot represents staining of a specimen from one patient (*N* = 22). (Fig. 6e).

### Expression of S100A9 and PD-L1 affects the survival of patients with PDAC

**Fig. 7:**
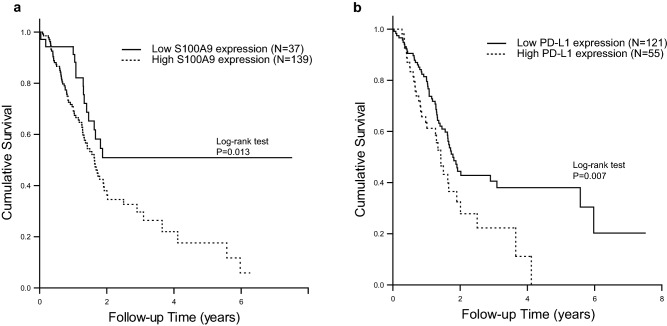
Kaplan–Meier survival curves generated based on S100A9 (**a**) and PD-L1 (**b**) expression. We obtained survival and immunohistochemistry staining data from 176 patients with PDAC from The Human Protein Atlas (https://www.proteinatlas.org/). Patients were divided into high and low expression groups based on the default setting on the website. The survival of each group was examined by Kaplan–Meier survival estimators, and the survival outcomes of the two groups were compared by log-rank tests. The patients with lower expression of S100A9 (**a**, *p* = 0.013, log-rank test) and PD-L1 (**b**, *p* = 0.007, log-rank test) in their PDAC samples had better survival than those with high expression

We next explored the Human Protein Atlas (https://www.proteinatlas.org/) database to identify correlations of S100A9 and PD-L1 expression with clinical outcomes of PDAC [33, 34]. The Human Protein Atlas database has data on the clinical outcomes and immunohistochemical staining of S100A9 and PD-L1 from 176 patients with PDAC. The clinical and pathological characteristics are described in Supplementary Table 4. Most of the patients included in the database had stage I or II disease (94.3%), and high expression of S100A9 predicted worse survival than low expression of S100A9 in this cohort (*p* = 0.017, log-rank test). In the cohort of 176 patients, we also found that higher levels of S100A9 expression correlated with higher levels of α-SMA, which is regarded as a marker of activated stellate cells[35, 36] (*p* < 0.001, chi-square test, Supplementary Table 4). The relationship between the expression of α-SMA and the survival of patients with PDAC was not significant (*p* = 0.144, log-rank test, Supplementary Fig. 1). Using univariate and multivariate Cox regression analyses of potential factors affecting patient survival in the cohort, we found that both patient age (*p* = 0.019) and S100A9 expression (*p* = 0.037) affected patient survival (Supplementary Table 5). With a cutoff for S100A9 and PD-L1 immunohistochemical expression determined by the default setting on The Human Protein Atlas website, high expression of S100A9 (Fig. 7a) and high expression of PD-L1 (Fig. 7b) correlated with worse overall survival than low expression of either marker in the cohort (*p* = 0.013 for S100A9; *p* = 0.007 for PD-L1, log-rank test).

## Discussion

Cancers are not composed of only neoplastic cells and also include fibroblasts, immune cells, endothelial cells and specialized stromal cells. These different cell types in the cancer microenvironment can be recruited by neoplastic cells to support their growth and dissemination. The interactions between cancer cells and stromal cells exert a major impact on neoplastic growth and progression [37, 38]. Understanding the interactions between cancer cells and their biological environment may be as important for future cancer therapies as understanding the cancer cell itself.

S100A9 is a calcium binding protein and may exist as a homodimer or heterodimer with S100A8 [39]. However, we were not able to detect significant changes in S100A8 expression in PDAC cells cocultured with PSCs by either quantitative PCR or Western blot (data not shown). One possible reason is that according to published reports, mature macrophages differentiated from monocytes initially express S100A8 and S100A9 but later lose S100A8 expression at inflammatory sites [40, 41]. S100A9 is expressed in many cell types, especially those of myeloid origin and can bind to cell surface receptors that trigger signaling pathways related to numerous cellular processes, including cell cycle progression, cell survival, proliferation, differentiation and migration [42]. S100A9 is also upregulated in many cancer types, including breast cancer, colon cancer, hepatocellular carcinoma, gastric cancer, colorectal cancer, non-small-cell lung cancer and cervical cancer [40, 43]. The expression of S100A9 is associated with a poor prognosis among non-small-cell lung cancer patients, which is consistent with our findings [43]. Three types of receptors have been reported for S100A9, including TLR4 [30, 44], receptor for advanced glycation end products (RAGE) [45] and extracellular matrix metalloproteinase inducer (EMMPRIN) [19]. It has been indicated that the level of S100A9 is increased at sites of inflammation, and that S100A9 can induce the migration of myeloid cells [44]. Several studies on different types of cancer cells have shown that S100A9 induces activation of NF-kB, and that knockdown of S100A9 expression decreases tumor invasion [17, 40, 46, 47]. S100A9 is abundant in myeloid cells and can be released upon activation [20, 42]. Our current study demonstrates that secretion of S100A9 is not limited to myeloid cells. We found that the secretion of S100A9 could be enhanced in pancreatic cancer cells when they were incubated with PSCs and that S100A9 acts as a chemoattractant to recruit monocytes.

In many cancers, the interaction of costimulatory molecules on T lymphocytes with the tumor-associated membrane-bound protein PD-L1 can lead to apoptosis of activated T cells [24]. Tumor-associated PD-L1 expression was reported to increase the apoptosis of activated T lymphocytes and assist cancer cells in escaping immune surveillance [24, 31, 48]. High expression of PD-L1 in pancreatic cancers is associated with a poor prognosis [25]. According to published reports, S100A8 and S100A9 can induce the expression of PD-L1 in macrophages [29, 49]. Therefore, we examined the effect of S100A9 on monocytes and found that S100A9 not only had chemotactic effects but could also induce surface expression of PD-L1 in monocytes. When exploring the Human Protein Atlas database to identify correlations of S100A9 and PD-L1 expression with clinical outcomes of PDAC, we found that high expression of S100A9 and PD-L1 was associated with worse overall survival than low expression of these proteins in patients with PDAC. In the specimens of 176 patients from the database, low expression levels of S100A9 correlated with reduced expression of PD-L1.

**Fig. 8:**
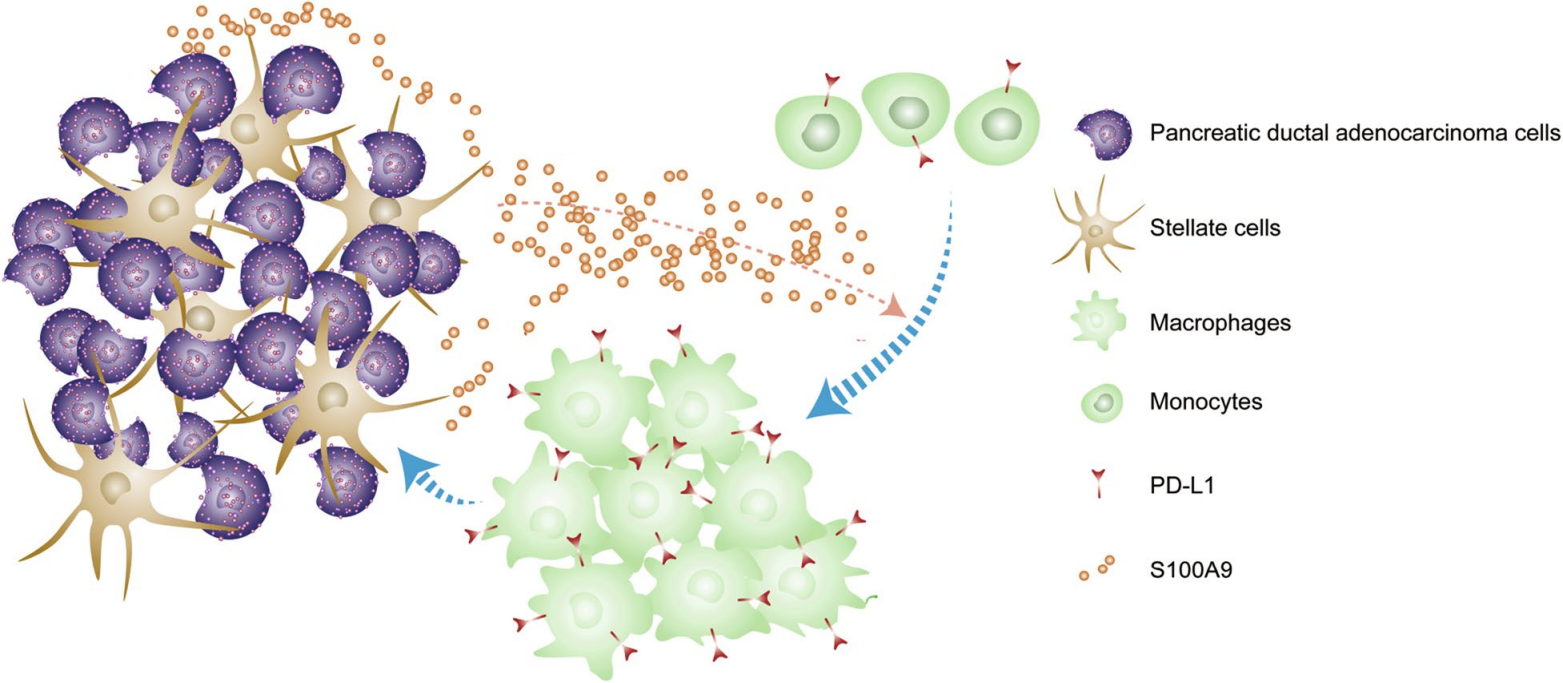
A proposed model summarizing the interaction between PDAC cells and their surrounding microenvironment. S100A9 expression can be induced in PDAC cells incubated with PSCs and acts as a chemoattractant for monocytes. S100A9 can also induce surface expression of PD-L1 on macrophages

In summary, in this study, we demonstrate that the secretion of S100A9 is not only limited to myeloid cells and may also be induced in pancreatic cancer cells incubated with PSCs. As indicated in Fig. 8, we found that S100A9 acts as a chemoattractant to recruit monocytes and induces the surface expression of PD-L1 on macrophages. Our results highlight that high expression of S100A9 and PD-L1 correlates with worse overall survival than low expression of these proteins in patients with PDAC.

### Supplementary Information

Below is the link to the electronic supplementary material.

Supplementary file1 (PDF 276 kb)

## Abbreviations

α-SMA: α-Smooth muscle actin
EMMPRIN: Extracellular matrix metalloproteinase inducer
GEO: Gene Expression Omnibus
IFN-γ: Interferon gamma
PBS: Phosphate-buffered saline
PCR: Polymerase chain reaction
PDAC: Pancreatic ductal adenocarcinoma
PD-L1: Programmed death-ligand 1
PSC: Pancreatic stellate cell
RAGE: Receptor for advanced glycation end products
S100A8: S100 calcium binding protein A9
S100A9: S100 calcium binding protein A9
shRNA: Short hairpin RNA
TLR4: Toll-like receptor 4

## Declarations

### Data availability

The datasets and materials used during the current study are available from the corresponding author on request. The publicly available microarray datasets GSE36775 and GSE9350 were downloaded from Gene Expression Omnibus (https://www.ncbi.nlm.nih.gov/geo/).
