# Supplementary material for: The role of S100A9 in the interaction between pancreatic ductal adenocarcinoma cells and stromal cells
Source: Cancer Immunol Immunother. 2021 Aug 10;71(3):705–18. doi: 10.1007/s00262-021-03026-y (PMC8854169; doi:10.1007/s00262-021-03026-y)
Supplement: Supplementary file 1 — Supplementary file1 (PDF 276 kb) [file 262_2021_3026_MOESM1_ESM.pdf]

Supplementary Information

Supplementary Table 1. Top 50 upregulated genes in pancreatic adenocarcinoma cells exposed to pancreatic stellate cells in the microarray dataset GSE36775. The microarray dataset was downloaded from Gene Expression Omnibus (GEO) at https://www.ncbi.nlm.nih.gov/geo/. We analyzed the dataset with GEO2R at https://www.ncbi.nlm.nih.gov/geo/geo2r/ to obtain log2-transformed fold change values and adjusted p-values for multiple comparisons.

Gene symbol	Log fold change	Adjusted p-value
SAA1	2.17	0.0018
FCGBP	1.94	0.00201
PIGR	1.91	0.00167
SAA2	1.91	0.00167
TFF3	1.77	0.0018
ATP10B	1.75	0.00243
HLA-DRA	1.69	0.00243
RNASE1	1.68	0.00165
AGR2	1.67	0.00243
S100A8	1.67	0.00165
RARRES1	1.65	0.0101
DMBT1	1.63	0.00165
CDC42EP5	1.57	0.00109
ASS1	1.54	0.00964
CFB	1.49	0.00667
CD74	1.45	0.00435
HLA-DPA1	1.42	0.00331
GBP2	1.36	0.00684
HSD17B2	1.33	0.00165
MUC1	1.33	0.00443
PSCA	1.33	0.01877
RARRES3	1.29	0.02695

CEACAM1	1.26	0.02399
PDZK1IP1	1.23	0.03226
PRR15L	1.23	0.00312
HLA-DRB6	1.18	0.00243
CAPN5	1.17	0.00697
CYP3A5	1.17	0.01538
LGALS4	1.16	0.00547
CD24	1.14	0.00667
PI3	1.14	0.04351
SERPINA3	1.14	0.04645
CCPG1	1.13	0.03932
HLA-DMA	1.13	0.0018
MB	1.13	0.0018
VSIG2	1.11	0.01812
WIPI1	1.11	0.0083
**S100A9**	**1.10**	**0.02042**
AZGP1	1.09	0.00167
KLHL24	1.08	0.02311
SAA4	1.08	0.00697
TRAPPC6A	1.07	0.0018
ALDH3B2	1.07	0.00264
PRSS1	1.06	0.01563
PLA2G10	1.04	0.00167
TSPAN1	1.03	0.00243

7

Supplementary Table 2. Top 50 upregulated genes in pancreatic cancer cell lines with high metastatic and growth potential under normoxic growth conditions in the microarray dataset GSE9350. The microarray dataset was downloaded from Gene Expression Omnibus (GEO) at https://www.ncbi.nlm.nih.gov/geo/. We analyzed the dataset with GEO2R at https://www.ncbi.nlm.nih.gov/geo/geo2r/ to obtain log2-transformed fold change values and adjusted p-values for multiple comparisons.

Gene symbol	Log fold change	Adjusted p-value
SOSTDC1	6.657	0.0000911
NTRK2	6.496	0.0001743
IL1R1	5.191	0.0012128
BCHE	4.9	0.0002484
PLAT	4.524	0.0002185
PTPRZ1	4.441	0.0005813
HLA-DRA	4.384	0.0000806
PHLDA1	4.241	0.0002185
MUC2	4.131	0.0015504
PTHLH	3.949	0.00111
MPPED2	3.946	0.0006904
SAGE1	3.933	0.0002284
PHLDA1	3.875	0.0013092
DUSP6	3.851	0.0012311
ALDH1A3	3.838	0.0006904
HS3ST1	3.675	0.0005678
NTRK2	3.603	0.0002302
DKK3	3.327	0.0007911
SLC6A15	3.284	0.0004901
PAK3	3.249	0.000486
GLUL	3.22	0.0000806

MME	3.173	0.0014382
PIK3AP1	3.172	0.0004212
PADI3	3.07	0.0002484
PAK3	3.065	0.0005813
DUSP6	3.048	0.001214
GLUL	3.017	0.0001232
GCNT3	3.013	0.0002187
ABCA1	2.973	0.0003563
VEGFC	2.934	0.00111
**S100A9**	**2.933**	**0.0003563**
PHLDA1	2.813	0.0007405
STC1	2.801	0.0015455
GLUL	2.79	0.0001284
TNFSF10	2.763	0.0012251
MPPED2	2.723	0.000737
ABCA1	2.627	0.0003407
GBP6	2.603	0.0006904
TNFSF10	2.403	0.0014056
LINC00673	2.275	0.000822
SLC6A15	2.259	0.0016643
CEP19	2.245	0.000822
CEP19	2.24	0.0011113
TIPARP	2.238	0.0002915
B4GALT6	2.218	0.0003563
NFKBIZ	2.198	0.0004531
IL18R1	2.192	0.0006686
IKZF2	2.178	0.0002187
EDNRA	2.115	0.001755
NRP2	2.049	0.00111

14

Supplementary Table 3. Primer pairs for real-time PCR

S100A8 F:	5'	GGGGAATTTCCATGCCGTCTAC	3'
S100A8 R:	5‘	CTGCCACGCCCATCTTTATCAC	3’
S100A9 F:	5'	TCAAAGAGCTGGTGCGAAAAGAT	3'
S100A9 R:	5'	CCTCGCCATCAGCATGATGAACT	3‘
Cyclophilin F:	5'	ATACGGGTCCTGGCATCTTGTC	3'
Cyclophilin R:	5'	GGTGATCTTCTTGCTGGTCTTG	3'

Supplementary Table 4. Clinical and pathological characteristics of the study cohort from The Human Protein Atlas website.

We obtained data from 176 patients with pancreatic ductal carcinoma via The Human Protein Atlas website (https://www.proteinatlas.org/). The patients were divided into high and low expression groups based on the default setting on the website. *p* values were calculated via * t-test, ** chi-square test, or *** Fisher's exact test. α-SMA: α-smooth muscle actin.

Characteristics	S100A9 level		*p* value
	Low<br>N=37	High<br>N=139	
Age (Years)	63.1±10.5	65.1±11	0.303*
Sex			
Male	24	72	0.156**
Female	12	67	
Stage			
I-II	32 (18.2%)	134 (76.1%)	0.036***
III-IV	4 (2.3%)	3 (1.7%)	
Not available	1 (0.6%)	2 (1.1%)	
α-SMA level			
Low	24	29	<0.001**
High	13	110	

Supplementary Table 5. Univariate and multivariate Cox regression analyses of potential factors affecting patient survival in 176 patients with pancreatic ductal carcinoma from the Human Protein Atlas database (https://www.proteinatlas.org/). α-SMA: α-smooth muscle actin.

	Univariate model		Multivariate model	
Factor	HR (95%CI)	*p* value	HR (95%CI)	*p* value
Age	1.03 (1.01-1.05)	0.006	1.03 (1-1.05)	0.019
Male	0.82 (0.54-1.24)	0.344		
Stage				
I	0.46 (0.21-0.99)	0.028		
II	2.37 (1.22-4.59)	0.005		
III	0.6 (0.08-4.32)	0.581		
IV	1 (0.25-4.12)	0.99		
S100A9 expression	2 (1.14-3.49)	0.009	1.82(1.04-3.21)	0.037
α-SMA expression	1.41 (0.89-2.24)	0.136		

Supplementary Figure 1.

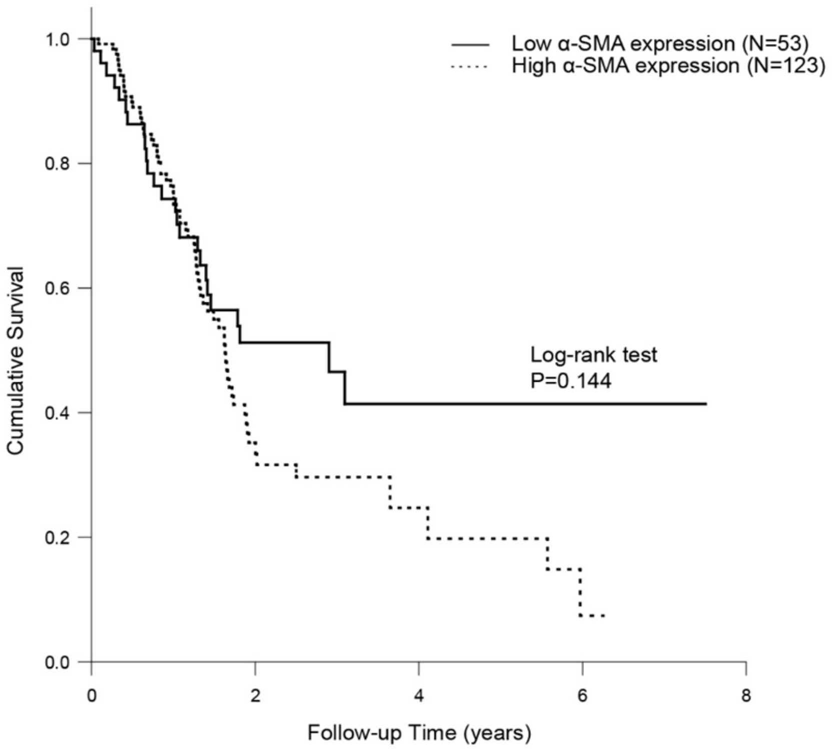

Kaplan-Meier survival curves according to α-smooth muscle actin (α-SMA) expression. We obtained survival and immunohistochemistry staining data from 176 patients with pancreatic ductal carcinoma from The Human Protein Atlas (https://www.proteinatlas.org/). Patients were divided into high and low expression groups based on the default setting on the website. The survival of each group was examined by Kaplan-Meier survival estimators, and the survival outcomes of the two groups were compared by log-rank tests. The expression of α-SMA did not have a significant effect on the survival of the patients with PDAC (p=0.144, log-rank test).

Supplementary Figure 2.

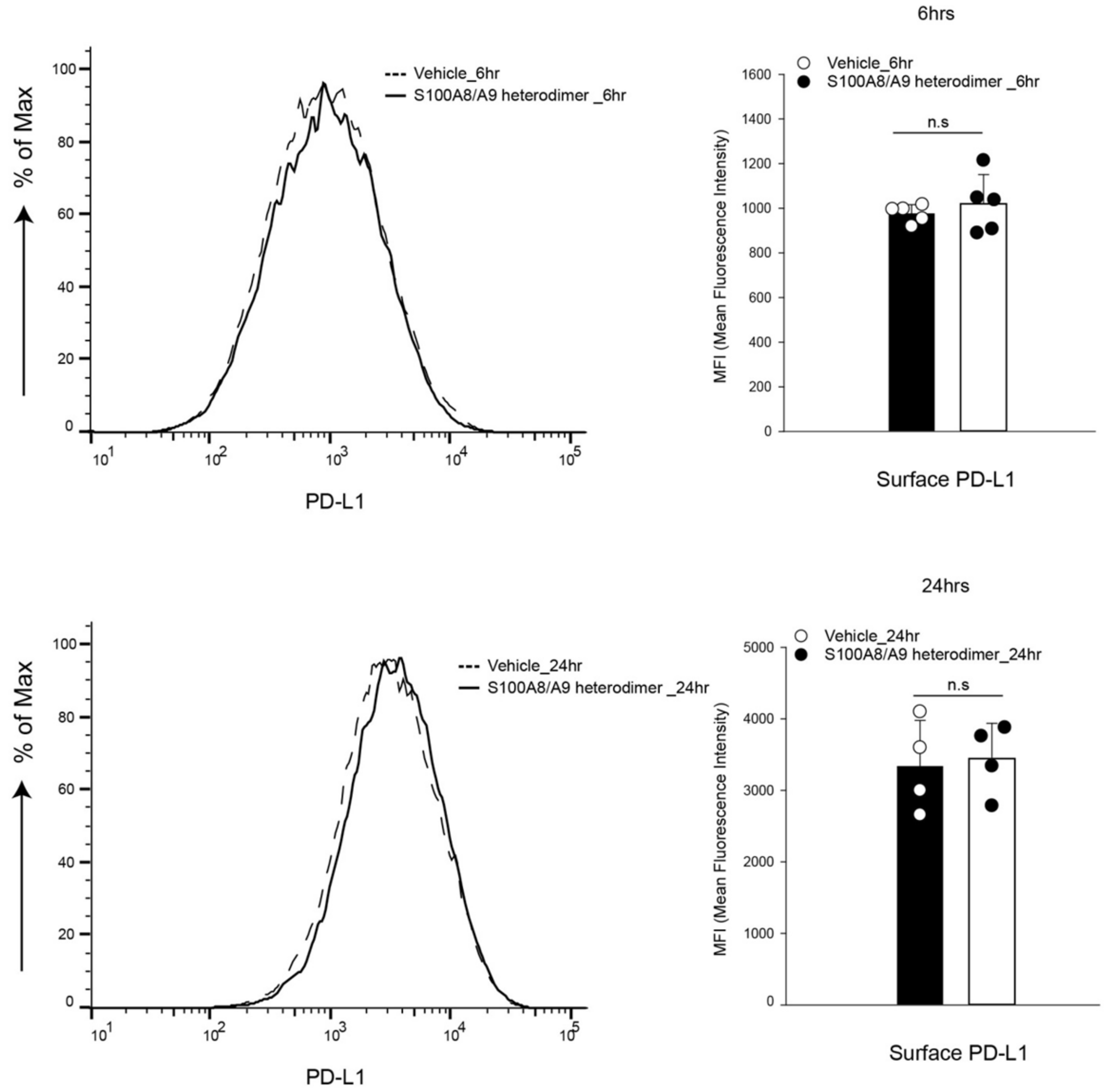

Supplementary Figure 2. Stimulation with S100A8/S100A9 heterodimers in monocyte-derived IFN-γ-primed U937 cells did not induce significant changes in the surface expression of PD-L1 in comparison with vehicle control.

We treated IFN-γ-primed U937 cells with recombinant S100A8/S100A9 heterodimers, and detected the surface expression of PD-L1 with flow cytometry. We found that treatment of IFN-γ-primed U937 cells with S100A8/S100A9 heterodimers for 6 and 24 hours did not induce significant changes in the surface expression of PD-L1 in comparison with vehicle control.
